# Signaling roles of phosphoinositides in the retina

**DOI:** 10.1194/jlr.TR120000806

**Published:** 2021-02-06

**Authors:** Raju V.S. Rajala

**Affiliations:** 1 Departments of Ophthalmology, Physiology, and Cell Biology, and Dean McGee Eye Institute, University of Oklahoma Health Sciences Center, Oklahoma City, OK 73104

**Keywords:** light activation, phosphoinositide kinases, phosphoinositide phosphatases, phosphoinositide binding proteins, CNG, cyclic nucleotide-gated, CNGA1, cyclic nucleotidegated channel subunit α1, CNTF, ciliary neurotrophic factor, EPO, erythropoietin, FYCO1, FYVE and coiled-coil domain autophagy adaptor 1, GPCR, G protein-coupled receptor, Grb14, growth factor receptor-bound protein 14, GSK3β, glycogen synthase kinase 3β, IGF, insulin-like growth factor, INPP5, inositol polyphosphate 5-phosphatase, IP_3_, inositol triphosphate, IR, insulin receptor, IRS, insulin receptor substrate, mTOR, mammalian target of rapamycin, OCRL, oculocerebrorenal syndrome of Lowe, PDGF, platelet-derived growth factor, PH, pleckstrin homology, PI, phosphatidylinositol, PIKfyve, FYVE-type zinc finger containing phosphoinositide kinase, PI3K, phosphoinositide 3-kinase, PI4K, phosphatidylinositol 4-kinase, PIP, phosphatidylinositol phosphate, PI(3)P, phosphatidylinositol 3-phosphate, PI(4)P, phosphatidylinositol 4-phosphate, PI(5)P, phosphatidylinositol 5-phosphate, PI(3,4) P_2_, phosphatidylinositol 3,4-bisphosphate, PI(3,5)P_2_, phosphatidylinositol 3,5-bisphosphate, PI(4,5)P_2_, phosphatidylinositol 4,5-bisphosphate, PI(3,4,5)P_2_, phosphatidylinositol 3,4,5-trisphosphate, PIPK, phosphatidylinositol phosphate kinase, PITP, phosphatidylinositol transfer protein, PKC, protein kinase C, PLC, phospholipase C, PTEN, phosphatase and tensin homolog, PTP1B, protein tyrosine phosphatase 1B, RA, Ras-associating, ROS, rod outer segment, RPE, retinal pigment epithelium, SH2, Src homology 2, SHIP, Src homology 2 domain-containing inositol polyphosphate 5-phosphatase, TRP, transient receptor potential, Vps, vacuolar protein sorting, VDAC, voltage-dependent anion channel

## Abstract

The field of phosphoinositide signaling has expanded significantly in recent years. Phosphoinositides (also known as phosphatidylinositol phosphates or PIPs) are universal signaling molecules that directly interact with membrane proteins or with cytosolic proteins containing domains that directly bind phosphoinositides and are recruited to cell membranes. Through the activities of phosphoinositide kinases and phosphoinositide phosphatases, seven distinct phosphoinositide lipid molecules are formed from the parent molecule, phosphatidylinositol. PIP signals regulate a wide range of cellular functions, including cytoskeletal assembly, membrane binding and fusion, ciliogenesis, vesicular transport, and signal transduction. Given the many excellent reviews on phosphoinositide kinases, phosphoinositide phosphatases, and PIPs in general, in this review, we discuss recent studies and advances in PIP lipid signaling in the retina. We specifically focus on PIP lipids from vertebrate (e.g., bovine, rat, mouse, toad, and zebrafish) and invertebrate (e.g., *Drosophila*, horseshoe crab, and squid) retinas. We also discuss the importance of PIPs revealed from animal models and human diseases, and methods to study PIP levels both in vitro and in vivo. We propose that future studies should investigate the function and mechanism of activation of PIP-modifying enzymes/phosphatases and further unravel PIP regulation and function in the different cell types of the retina.

Phosphatidylinositol (PI) is a minor constituent (∼0.5–1%) of the total phospholipid pool in the cell membrane (1). Phosphatidylinositol consists of a head group of D-*myo*-inositol and a backbone of the trihydroxy alcohol, glycerol, in which the C1 and C2 positions of glycerol are occupied with two fatty acids (Fig. 1). The free-OH groups (3, 4, and 5) in the inositol head group undergo phosphorylation by specific phosphoinositide kinases. These phosphorylated phosphatidylinositol phosphates are collectively called phosphoinositides (PIPs).

**Fig. 1:**
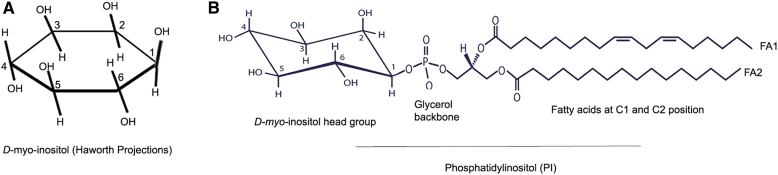
Structure of phosphatidylinositol (PI). D-*myo*-inositol presented as a Haworth projection (A). Phosphatidylinositol contains D-*myo*-inositol attached to a glycerol backbone and two fatty acids attached to the C1 and C2 positions of the glycerol (B).

The intracellular pools of PIPs are dynamically converted from one form to the other through the action of specific phosphoinositide kinases, and phosphoinositide phosphatases can generate seven distinct PIPs (1, 2, 3) (Fig. 2). These molecules include PI(3)P, PI(4)P, PI(5)P, PI(3,4)P_2_, PI(3,5)P_2_, PI(4,5)P_2_, and PI(3,4,5)P_3_. Of these PIPs, the D3-phosphoinositides include PI(3)P, PI(3,4)P_2_, and PI(3,4,5)P_3_; the D4-phosphoinositides include PI(4)P and PI(4,5)P_2_; and the D5-phosphoinositides include PI(5)P and PI(3,5)P_2_. The D3-PIPs are formed by the action of the phosphoinositide 3-kinases (PI3Ks), which use PI, PI(4)P, and PI(4,5)P_2_ to generate PI(3)P, PI(3,4)P_2_, and PI(3,4,5)P_3_. The D4-PIPs are formed by the action of the PI4 kinases (PI4Ks), which use PI and PI(5)P to generate PI(4)P and PI(4,5)P_2_. The D5-PIPs are formed by the action of the enzyme PI5K, which uses PI and PI(3)P to generate PI(5)P and PI(3,5)P_2_ (1, 3). These seven distinct PIPs are present in all mammalian cells (4), and their formation is also present in the retina/photoreceptor cells (5, 6, 7, 8). The D3-, D4-, and D5-phosphoinositides regulate cytoskeletal organization, membrane fusion and budding, ciliogenesis, vesicular transport, and signal transduction (9, 10). PI(3)P is involved in the vesicle export from the Golgi. PI(4,5)P_2_ is involved in exocytosis, cytoskeletal regulation, and regulation of phospholipase D/A_2_. PI(3,5)P_2_ regulates Golgi, lysosomal/endosome trafficking, and osmoprotection. PI(3,4,5)P_3_ is involved in cell survival, solute transport, and regulation of ARF/Rac protein (1, 9).

**Fig. 2:**
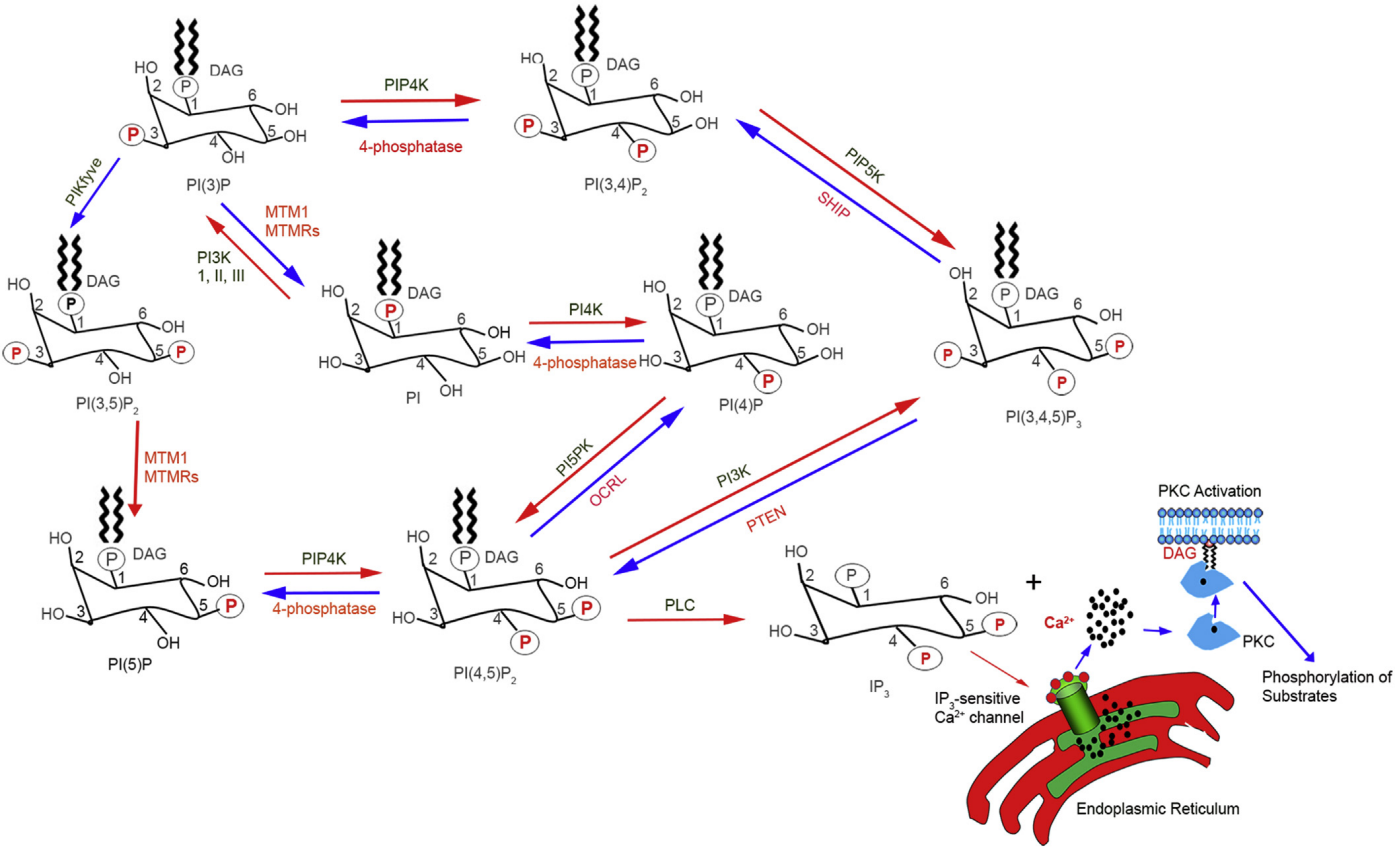
Generation of seven distinct phosphoinositides. The inositol head group contains several free hydroxyls that undergo phosphorylation by phosphoinositide kinases and dephosphorylation by phosphoinositide phosphatases. Phosphorylation of free hydroxyls at the 3, 4, 5 positions facilitates the generation of seven PPIs. PI(4,5)P_2_ undergoes hydrolysis by phospholipase C (PLC) generates two-second messenger signaling molecules, diacylglycerol (DAG) and inositol 1,4,5-trisphosphate (IP_3_). IP_3_ binds to IP_3_-sensitive Ca^2+^ channels on the endoplasmic reticulum and mobilizes the release of Ca^2+^. Both, DAG and Ca^2+^ activate protein kinase C (PKC). Activated PKC phosphorylates its downstream target proteins. MTMl, myotubularin l (3’phosphatase); MTMRs, myotubularin-related phospholipid phosphatase; PI(3)P, phosphatidylinositol 3-phosphate; PI(4)P, phosphatidylinositol 4-phosphate; PI(5)P, phosphatidylinositol 5-phosphate; PI(3,4)P_2_, phosphatidylinositol 3,4-bisphosphate; PI(3,5)P_2_, phosphatidylinositol 3,5-bisphosphate; PI(4,5)P_2_, phosphatidylinositol 4,5-bisphosphate; PI(3,4,5)P_3_, phosphatidylinositol 3,4,5-trisphosphate. PIP4K, phosphatidylinositol 5-phosphate 4-kinase; PI3K, phosphoinositide 3-kinase; PIP5K, phosphatidylinositol 4-phosphate 5-kinase; PTEN, phosphatase and tensin homolog; SHIP, Src homology 2 (SH2) domain-containing inositol polyphosphate 5-phosphatase; PIKfyve, FYVE-type zinc finger containing phosphoinositide kinase; OCRL, Oculocerebrorenal inositol polyphosphate-5-phosphatase; PI4K, phosphatidylinositol 4-kinase.

There are many excellent reviews on phosphoinositide kinases, phosphoinositide phosphatases, and PIPs in general. This review describes the recent studies and advances in PIP lipids and their signaling in the retina. The PIP lipids from vertebrate (e.g., bovine, rat, mouse, toad, zebrafish) and invertebrate [e.g., *Drosophila*, limulus (horseshoe crab), squid] retina are discussed. The importance of PIPs revealed from animal models and human diseases and methods to study PIP levels in vitro and in vivo are also described.

## Phosphoinositides in the retina

In 1981, Anderson and Hollyfield (11) reported that light stimulates the incorporation of inositol in the vertebrate retina. Light has also been shown to stimulate the generation of PIs in horizontal cells of the retina (12, 13). Subsequent studies showed that light adaptation of bovine retinas in situ stimulates PI synthesis in retinal rod outer segment (ROS) membranes in vitro (5). The work described in 1983 and 1984 by Anderson and colleagues revealed a functional significance in photoreceptor horizontal cell synapses (12, 13). Light stimulates the PI metabolism in these cells, and these PIPs are important for synaptic ribbon formation, glutamate release, and signaling (12, 13).

Phosphatidylinositol is a comparatively large component of the membranes of most cells in metazoans ranging from 4 to 20 mol% of total phospholipid (1, 14), and analysis of phosphatidylinositol in six different mammalian species shows 4.4–6.4% mol% of total retina phospholipid (14, 15, 16, 17). Studies have shown that retinal ROS membranes contain lower levels of PI than do retinal pigment epithelium (RPE) cells (18) and ER membranes isolated from bovine retinas (19). Recently, Wensel’s laboratory reported that ROSs and fragments of inner segments contain PI(3)P at 0.0035 mol% and PI(4)P and PI(4,5)P_2_ on the order of 0.04 mol%, 10-fold higher than the levels of PI(3)P of total phospholipid (14).

Phototransduction is modulated by phosphoinositides within photoreceptors (5, 20), which increase phospholipase C (PLC) activity in the outer segment membranes, increase the uptake of radiolabeled inositol and phosphoinositide turnover in photoreceptor cells (20, 21), and release inositol 1,4,5-trisphosphate (IP_3_) from the retina (22). Furthermore, IP_3_ receptors (23) and PLC (24, 25) are expressed in the outer segments. In invertebrates, phototransduction is mediated through PLC activation, while in the vertebrate retina, the phototransduction cascade is mediated through rhodopsin activation and subsequent hydrolysis of cGMP (26, 27). Interestingly, in the intrinsically photosensitive retinal ganglion cells, PLC-mediated hydrolysis is activated by the photopigment, melanopsin, which couples Gq to open the transient receptor potential (TRP) channels (28), suggesting evolutionarily conserved pathways that use PI(4,5)P_2_ as a substrate for the modulation of phototransduction.

One of the potential functions of PLC is involvement in the translocation of arrestin from inner to outer segments of photoreceptor cells (29). Furthermore, activators of the downstream effector of PLC, protein kinase C (PKC), and PLC facilitate arrestin translocation to outer segment membranes, independent of light (29). Physiological studies also show that phototransduction is modulated by PIPs (14, 30). The cone cyclic nucleotide-gated (CNG) (31) and olfactory (32) channels are known to be inhibited by PI3K-generated PI(3,4,5)P_3_.

It has been suggested that phosphoinositides play an important role in photoreceptor cell processes, such as disk morphogenesis, endocytosis, exocytosis, membrane budding, endosomal sorting, and post-Golgi vesicle trafficking. In rhodopsin trafficking, the involvement of PI(3)P and PI(4,5)P_2_ has been demonstrated (33, 34). The actin-nucleating proteins Arp2 and Arp3 are demonstrated to be involved in basal disc extensions (34), and local pools of PI(4,5)P_2_ may be important for their function (35, 36).

## Metabolism of phosphoinositides in the retina

There is an active PI metabolism in the vertebrate retina and ROSs (6, 7, 8, 12, 13, 20, 21, 22, 25, 37, 38, 39, 40, 41, 42, 43, 44, 45, 46, 47, 48, 49, 50, 51). Light has been shown to stimulate several enzymes that are involved in PI metabolism, such as class I PI3K (7, 52), class III PI3K (53, 54), PI synthetase (5), DAG kinase (6), phosphatidylethanolamine N-methyltransferase (55), phospholipase A2 (56), phospholipase D (57), PLC (58, 59, 60, 61), PKC (62), lipid phosphate phosphatase (63), and DAG lipase (37). Light also modulates the second messengers generated in the retina from DAG, PC, and PA (37). Further, light activates proteins regulated by insulin signaling (64, 65, 66, 67).

## Phosphoinositide kinases and phosphatases

Forty-seven genes encode 19 phosphoinositide kinases and phosphatidylinositol phosphate (PIP) kinases (PIPKs) and 28 PIP phosphatases in mammals (2). The action of the lipid kinases, PI3K, PI4K, and PI5K, and PI3-, PI4- and PI5-specific lipid phosphatases can generate seven distinct phosphoinositide lipids in mammalian cells (1, 2, 3). The phosphoinositide kinase isoforms are divided into three major families: the PI 3-kinases (PI3Ks), PI 4-kinases (PI4Ks), and PIPKs (68).

## Phosphoinositide 3-kinases

**Fig. 3:**
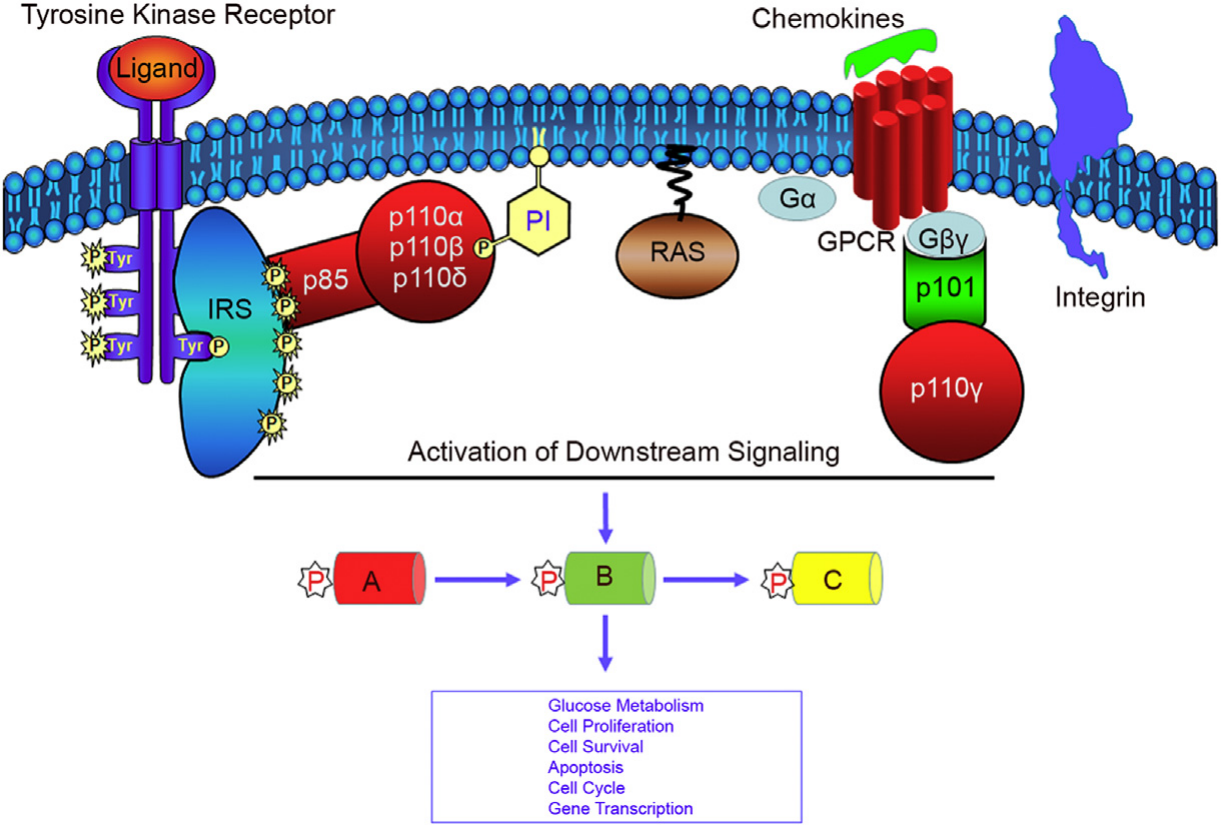
Activation of phosphoinositide 3-kinases. PI3Ks are activated through tyrosine kinase receptor activation, interaction with RAS, through GPCR activation, or are integrin-mediated. The PIPs generated at the membrane attract phospholipid-binding proteins in the cytosol, activate the downstream signaling (A–C), and regulate various aspects of cellular functions. In the case of the IR, IRS adaptor proteins bind to the IR by facilitating a platform for the binding of multiple PI3K molecules and amplify the signal.

The phosphoinositide 3-kinases have been broadly divided into four classes: class I, class II, class III, and class IV. Members of class I PI3K enzymes are heterodimers and consist of a p110 kDa catalytic subunit and a p85 regulatory subunit that contains two Src-homology regions capable of binding to phosphotyrosine sequences on the growth factor receptors (10). The growth factors, such as platelet-derived growth factor (PDGF), insulin, insulin-like growth factor (IGF)-1, and nerve growth factors, bind to its cognate receptor tyrosine kinase(s) and activate class I PI3Ks (69) (Fig. 3).

In mammals, class I PI3K-p110 catalytic subunits are encoded by four genes: *Pik3ca*, *Pik3cb*, *Pik3cg*, and *Pik3cd*, which are referred to as PI3Kα, -β, -γ, and -δ (70). The expression profile of these genes in tissues is not uniform. Ubiquitous expression of *Pik3ca* and *Pik3cb* genes has been demonstrated, whereas leukocytes specifically express *Pik3cg* and *Pik3cd* genes (70). *Pik3cg* is also expressed in cardiac tissues (71). All of the PI3Kα, -β, -γ, and -δ-p110 subunits have the phosphatidylinositol kinase domain, a catalytic domain, a C2 lipid-binding domain, and a GTPase Ras domain; these proteins all have a significant homology at the N-terminal end of the molecule (72). In mammalian cells, the catalytic subunits of p110α, p110β, and p110δ are associated with any of five different regulatory subunits, p85α, p85β, p55γ, p55α, and p50α, referred to as “p85 subunits,” for phosphorylation of the lipid substrates (73). The p85 regulatory subunits are encoded by three genes, *Pik3r1*, *Pik3r2*, and *Pik3r3* (73). *Pik3r1* encodes the p85α, p55α, and p50α subunits. Both regulatory subunits, p85α and p85β, are universally expressed in all tissues (72), including retina (74), whereas p55γ is specifically expressed in brain tissues (75). The expression of p50α and p55α has been demonstrated in fat, muscle, liver, and brain tissues (76, 77). The regulatory p85 subunits interact with a unique domain present at the N-terminal end of the p110 catalytic subunits (78). In addition to the Src homology 2 (SH2) domain, the p85 regulatory subunit also contains an SH3 domain, which is capable of binding to proline-rich sequences and also contains a region with high conservation to the breakpoint cluster region (78). Extensive studies on class I PI3K have been carried out in the retina, especially in photoreceptor cells. Functionally, class I PI3K is essential for cone photoreceptor structure and function but not for rod photoreceptor cell survival (74, 79, 80).

Research also indicates that class IA PI3Kβ activation is regulated both by the G protein-coupled receptors (GPCRs) and receptor tyrosine kinases (81). The class I PI3Kγ lacks the p85-binding motif; hence, it interacts with p101 (82) and p84/87 adaptor proteins for regulation (83, 84). The class IB PI3Kγ is activated through GPCR signaling (Fig. 3). Upon GPCR activation by the ligand(s), heterotrimeric G protein composed of α-, β-, and γ-subunits binds to the GPCR (85). The α-subunit of G protein in its bound GDP readily exchanges with the cytosolic GTP, and upon GPCR activation dissociates from the rest of the βγ-subunits and activates the downstream effector molecules (86). The free G_βγ_-subunits of heterotrimeric G proteins, mostly the G_i_ subtype, activate PI3K signaling (71, 87) (Fig. 3). The PI3K family of lipid kinases has also been shown to be stimulated by chemokines in regulating the chemotaxis of inflammatory T lymphocytes, eosinophils, neutrophils, and macrophages, and PI3K signaling regulates the chemokine-activated cell migration (88).

The structure and function of class II PI3Ks are distinct from those of class I PI3Ks. The class II PI3Ks have a C-terminal C2 domain, which lacks a critical aspartate residue that coordinates the binding of Ca^2+^. The class II PI3K binds to lipid in a Ca^2+^-independent manner (89, 90). The class II PI3K consists of three catalytic isoforms, which include C2α, C2β, and C2γ, but is distinct from class I and III, as they do not have regulator subunits (89, 90). The class II enzyme catalyzes the conversion of PI to PI(3)P and PI(4)P to PI(3,4)P_2_ (89, 90). PI(3,4)P_2_ has been shown to play a role in the invagination step of clathrin-mediated endocytosis (91). The class II PI3K C2α and C2β isoforms are ubiquitously expressed in all tissues, but the expression of the C2γ isoform is restricted to hepatocytes (91). To date, there are no available studies of class II PI3K in the retina.

The structure and function of class III PI3K are distinct from those of class I and class II PI3K. Class III PI3K, also known as vacuolar protein sorting (Vps)34, was first identified in budding yeast, *Saccharomyces cerevisiae*, and screens for proteins involved in the regulation of vesicle-mediated Vps (92). Several PI(3)P binding proteins have been identified, and all are involved in protein trafficking (93). The crystal structure of Vps34 has been solved (94). Class III enzymes, which phosphorylate only PI, are heterodimers of a catalytic subunit associated with the serine/threonine-protein kinase adaptor subunit that is required for membrane recruitment (9, 10). The class III PI3K, Vps34p, is responsible for producing the majority of the cellular PI(3)P and is involved in protein trafficking through the lysosome (93). Class III PI3K Vps34 is closer to class I PI3K in terms of heterodimers of catalytic (Vps34) and regulatory (Vps15, a 150 kDa protein) subunits (95). Class III enzyme-generated PI(3)P is mainly involved in the trafficking of vesicles and proteins (96). Some studies show that PI(3)P regulates immune cell function and phagocytosis (97, 98). Class III PI3K plays an important role in rod, bipolar, and RPE cell functions in the retina (14, 53, 99, 100).

Class IV is a collection of enzymes, which include ataxia-telangiectasia mutated, ataxia telangiectasia and Rad3-related, DNA-dependent protein kinase, and mammalian target of rapamycin (mTOR). These enzymes are occasionally referred to as the class IV PI3Ks. Unlike class I, II, and III PI3Ks, which are lipid kinases, class IV PI3Ks are protein serine/threonine kinases (101). However, in vitro, class I PI3K has been shown to have protein kinase activity (102).

## Insulin receptor-regulated class i PI3K in photoreceptor cells

In 1997, class I phosphoinositide 3-kinase (PI3K) was reported to be responsible for the generation of D3-PIPs in retinal ROSs (7). Furthermore, tyrosine phosphorylation in vitro was shown to stimulate the PI3K activity in isolated bovine retinal outer segment membranes (8). The class I PI3K is composed of two subunits, a p110 catalytic subunit and a p85 regulatory subunit (10). With the application of p85 regulatory subunits that contain both N-terminal and C-terminal SH2 domains, Rajala and Anderson (103) identified that the insulin receptor (IR) in the retina/ROS is the receptor that regulates P13K activity. The authors also observed that in vitro tyrosine phosphorylation enhanced the phosphorylation of the IR, which results in the recruitment of the p85-N-SH2 domain, binds to the tyrosine-phosphorylated IR, and activates PI3K (103).

**Fig. 4:**
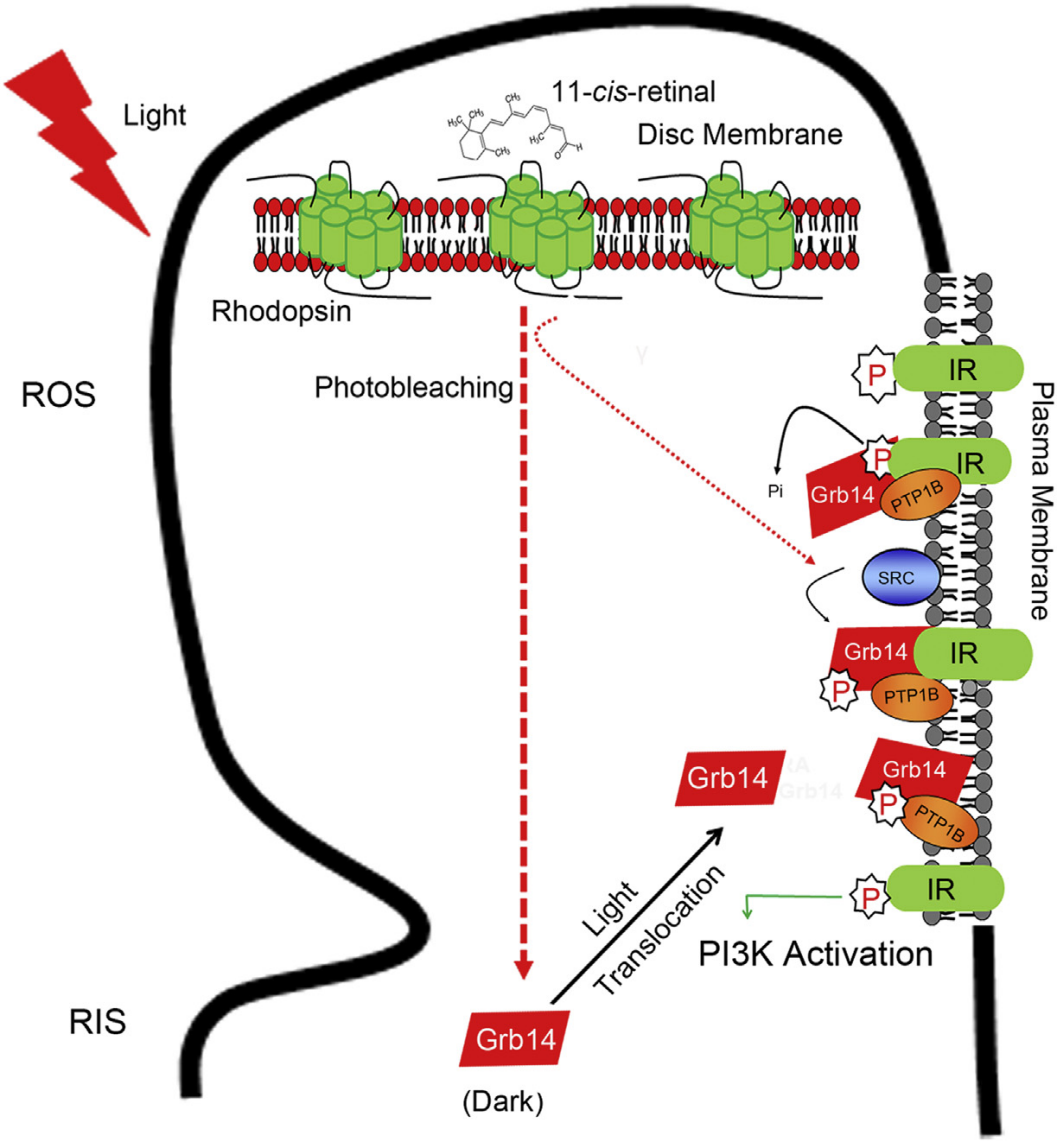
Noncanonical IR signaling-mediated activation of PI3K in the retina/photoreceptor cells. The IR in the retina is constitutively activated. However, the activated (phosphorylation) state of the IR is light-dependent. Under a dark-adapted state, the IR undergoes dephosphorylation by PTP1B and keeps the IR in an inactive state. Upon illumination, rhodopsin activation facilitates the translocation of Grb14 from inner segments to outer segments, where it undergoes a light- and rhodopsin-dependent phosphorylation by a nonreceptor tyrosine kinase, Src. Phosphorylated Grb14 binds to PTP1B and inhibits its activity, thus preserving the phosphorylation of the IR, which can bind to PI3K and generate PIPs. RIS, rod inner segments; Pi, inorganic phosphate; P, phosphorylation.

In rod photoreceptors, PI3K activation is mediated through light-dependent tyrosine phosphorylation of the IR (52, 104) (Fig. 4). The IR activation has been shown to be independent of G protein transducin activation and dependent on the photobleaching of rhodopsin (52). This IR/PI3K activation is a noncanonical rhodopsin activation (105) (Fig. 4). In the retina, the IR is constitutively phosphorylated (106, 107). However, the phosphorylation state of the IR is regulated through dark and light adaptation (108). In dark-adapted conditions, the IR is in the inactive state because of increased protein tyrosine phosphatase 1B (PTP1B) activity, which dephosphorylates the IR and results in the reduced association of PI3K with the IR (108). Upon rhodopsin activation, adaptor protein growth factor receptor-bound protein 14 (Grb14) localized to rod inner segments in the dark translocates to the outer segments in light and undergoes tyrosine phosphorylation by a nonreceptor tyrosine kinase, Src, in a rhodopsin-dependent manner (67, 109) (Fig. 4). The Src phosphorylation was abolished in animals deficient in the photobleaching of rhodopsin (109). The tyrosine-phosphorylated-Grb14 binds to PTP1B and inactivates its activity (Fig. 4). Thus, the IR becomes active, which then activates PI3K and promotes photoreceptor survival (108, 109, 110) (Fig. 4). Conditional deletion of the IR in rod photoreceptors resulted in stress-induced photoreceptor degeneration (111). The serine/threonine kinase Akt2, the downstream effector of IR, and PI3K deletion result in stress-induced photoreceptor degeneration (112). The proteins that regulate IR singing pathway in rods are also expressed in cones (113, 114). Interestingly, deletion of the IR in cones resulted in cone degeneration without added stress (113).

## Interaction between phosphoinositides and phospholipid-binding proteins

Several pleckstrin homology (PH) domain-containing proteins are expressed in the retina. PIPs regulate these proteins directly or indirectly. These proteins include PH domain retinal protein 1 (PHR1) (115, 116), Evectin-1 (117), Grb14 (118), IR substrate (IRS)-1 and IRS-2 (106, 119, 120), and three isoforms of Akt (Akt1, Akt2, and Akt3) (66, 106, 112). Evectin-1 is involved in the trafficking of post-Golgi membranes in photoreceptor cells (117). The PH domain retinal protein 1 PH domain is identical to serine/threonine kinase Akt and does not bind to inositol phosphates but interacts with transducin Gβγ subunits (115, 116). In the retina, the Akt-PH domain binds to PIPs (66). The PH domain of Akt binds to the cytoskeletal protein myosin II (121). This protein is mutated in patients with Usher syndrome (122). However, the interaction between myosin and Akt has not been tested in the retina. PH domain and leucine-rich repeat protein phosphatase (PHLPP) and PH domain and leucine-rich repeat protein phosphatase-like (PHLPPL) are PH domain-containing proteins known to dephosphorylate Akt isoforms (123, 124) and are also expressed in the retina (125, 126, 127).

## Downstream effects of PI3K signaling in the retina

In vivo, light stimulates the tyrosine phosphorylation of the IR, which results in the recruitment of PI3K and subsequent activation of Akt, the downstream effector of PI3K regulated through PI3K-generated PIPs (104). The addition of insulin as the ligand for the IR also stimulates the tyrosine phosphorylation of the IR, which results in the activation of PI3K ex vivo (66, 103). In the retina, light stress induces the phosphorylation of the IR, which results in the activation of the PI3K/Akt pathway through increased tyrosine phosphorylation of the IR (111). Addition of insulin growth factor-1, the ligand for IGF-1R, ex vivo to retinas stimulates the tyrosine phosphorylation of the IGF-1R, which results in the activation of the PI3K/Akt pathway (128). Interestingly, light stress, but not physiological light, stimulates the tyrosine phosphorylation of the IGF-1R that results in the activation of the PI3k/Akt downstream signaling pathway (128). In the retina, PI3K activation stimulates serine/threonine kinase Akt in vitro and in vivo (66, 106, 112).

Akt kinase exists as three isoforms: Ak1, Akt2, and Akt3. All three isoforms are expressed in retina and rod photoreceptor cells (66, 106). Membrane binding of Akt1 is mediated through its PH domain binding to PI3K-generated PIPs (66). In transgenic *Xenopus laevis*, the PH domain fused to GFP under the control of the *Xenopus* opsin promoter binds to PIPs in a light-dependent manner (66). In dark-adapted conditions, the actin exists as a stress fiber phenotype. Upon light-illumination, reorganization of the actin cytoskeleton was found to colocalize with PIPs in a *X. laevis* model (66). The expression of a mutant version of the PH domain in which the arginine 25 residue is substituted with cysteine failed to bind to PIPs (66).

The IR/PI3K/Akt signaling proteins are also expressed in the rod inner segments. These proteins may play an important role in cellular signaling events (105). One of the functions of PI3K-activated Akt in the inner segment is to regulate hexokinase 2 as it interacts with mitochondria in the photoreceptors (125). Growth factor-mediated activation of Akt was demonstrated to increase the association of hexokinase 2 with mitochondria in normal tissues and cells (129). Akt activation inhibits the dissociation of hexokinase 2 from mitochondria and is the primary event in the induction of apoptosis (125, 129, 130, 131). The mechanism of interaction between hexokinase 2 and mitochondria is that Akt phosphorylates glycogen synthase kinase 3β (GSK3β), which renders GSK3β inactive (132, 133). In the absence of Akt activation, GSK3β is active and phosphorylates the voltage-dependent anion channel (VDAC) on serine 51 that disrupts the binding of hexokinase 2 to the VDAC (134). The PI3K-generated PIP-activated Akt prevents the release of cytochrome c from mitochondria and inhibits apoptosis (129, 135). Retinal photoreceptors are postmitotic neurons and apoptosis is detrimental; therefore, the PIP-activated Akt promotes photoreceptor survival (112, 125).

## Interaction between the rod cng channel and PI3K in rod photoreceptor cells

Class I PI3Kγ lacks a p85-binding motif. Hence, it interacts with p101 (82) and p84/87 adaptor proteins for regulation (84, 136). Class I PI3Kγ is known to be regulated through Ras proteins (137) (Fig. 3). Ras belongs to a family of related proteins called small GTPases (138). These proteins have a Ras-associating (RA) domain, and PI3K interacts with this RA domain (139). In photoreceptor cells, the C-terminal region of the rod CNG channel subunit α1 (CNGA1) has 50–70% tertiary structural homology with Ras proteins (140). This domain has been named the Ras-like domain (140). In rod photoreceptor cells, PI3Kγ activation occurs through the interaction of its RA -domain with the Ras-like domain of CNGA1 (141). The interaction of PI3Kγ with CNGA1 does not affect the channel physiology. However, PI3Kγ uses CNGA1 as an anchor to achieve a close vicinity to its substrates to generate PIPs (141).

## PI3K KO phenotypes in the retina and rpe

In the retina, loss of p85α does not affect the overall morphology, but decreased PI3K activity associated with the IR is observed (142). Conditional deletion of the p85α (*pik3r1*) regulatory subunit in rod photoreceptor cells does not affect the structure of the retina (74). Mice with conditional deletion of p85α exhibited a slight delay in recovery kinetics and a delay in the translocation of rod arrestin from the inner segments to the outer segments (74). The absence of the disease phenotype in mouse rods lacking p85α could be explained by the expression of p85β in p85α KO mice (74). Interestingly, the deletion of the p85α-subunit of PI3K in cones results in age-related cone degeneration (79). The surviving cone-terminals of the cone-p85α KO mice exhibit progressive disorganization of synaptic structures (79). The loss of p85α in cones does not affect rod structure and function (79). Conditional deletion of the catalytic subunit of PI3K, p110α, in cones also resulted in cone degeneration (143). These studies highlight that PI3K signaling is indispensable for cone photoreceptor survival. These findings also suggest that other PIP-signaling pathways may regulate rod survival.

Conditional deletion of the class III PI3K *Vps34* gene in rods results in a failure in the fusion of endosomal and autophagy-related membranes with lysosomes that prompts the buildup of anomalous membrane structures (53). These mice have normal structure and function and trafficking of rhodopsin to the outer segments; however, the mice experience progressive rod degeneration by 12 weeks of age (53). The rod degeneration accompanying *Vps34* gene deletion is much faster than that of rods lacking autophagy genes, highlighting that PI(3)P is required for endosome recycling and other pathways that are necessary for rod photoreceptor survival (53). Vps34 has recently been shown to be essential for on-bipolar cell survival. Loss of this enzyme in these cells results in a significant loss of structure and function (99). This study further highlights that PI(3)P is necessary for the fusion of autophagosomes with lysosomes and maturation of late endosomes, and PI(3)P is needed for the maintenance of on-bipolar cells health (99).

In RPE, PI(3)P is essential for the fusion with autophagosomes, lysosomes, and phagosomes (100). These findings highlight that PI(3)P is essential for RPE cell health. In cone photoreceptors, the ablation of Vps34 resulted in an age-related cone degeneration (R. V. S. Rajala, unpublished observations).

## Phosphatidylinositol phosphate kinases

Phosphatidylinositol 4-kinases (PI4Ks) make PI(4)P from phosphatidylinositol (3). PI(4)P is an important molecule for the generation of other phosphoinositides involved in signaling, such as PI(4,5)P_2_, and is the substrate for the generation of second messengers, such as IP_3_ and DAG, through the action of PLC (144). PI(4,5)P_2_ also serves as a substrate for PI3K for the generation of PI(3,4,5)P_3_ (10, 145). PIPKs phosphorylate the D5 position of inositol on PI(4)P and the D4 position of PI(5)P to generate PI(4,5)P_2_ (146). Numerous PIPKs have been identified, purified, and cloned (147, 148, 149), and are separated into type 1 and type II based on their biochemical and immunological characteristics (10, 150). Based on substrate specificity, PIPK1 (type 1) phosphorylates PI(4)P and PIPKII (type II) phosphorylates PI(5)P (151). The mechanism of activation of PIPKs is not completely understood. Type I PIPK is activated by GTPγs (152), small G proteins (153), PA (154), heparin, and spermine (155). Spermine and PA do not affect PIPKII activity, but heparin inhibits PIPKII activity (155). Tyrosine phosphorylation has been shown to influence the activity of PIPKs (156).

In most cells, the PI(4,5)P_2_ is generated through type I PIPKs and global KOs of the α, β, and γ isoforms of this kinase have been produced (157). This isoform is highly expressed in the retina (158) and other neurons (159, 160). Early postnatal mortality was reported when this kinase was deleted (161). The mouse genes that encode PIPKs are *Pip4k2a*, *Pip4k2b*, *Pip4k2c*, *Pip5k1a*, and *Pip5k1c*; all of these were detected in the retinal mouse proteome (162).

In bovine ROS membranes, type II PIPK activity is regulated by tyrosine phosphorylation (163), and PI(5)P serves as a substrate for the synthesis of PI(4,5)P_2_ (163). In rodents and transgenic frog retina, PIPKIIα activity is stimulated by light, and the membrane binding of PIPKIIα to ROS proteins is tyrosine phosphorylation-dependent (164). PI(4,5)P_2_ is a critical PIP and regulates several key biological processes, such as actin cytoskeletal organization, endocytosis, exocytosis, modulation of ion channels, gene expression, angiogenesis, vesicular transport, cell migration, and nuclear functions (165). PI(4,5)P_2_ has been shown to activate cGMP-phosphodiesterase in ROS membranes, which results in the inhibition of ion influx through CNG channels (30, 166). In mammalian retina, the rod CNG channels, KCN1, TPR channels, and Na^+^-Ca^2+^ exchange are known to be regulated by PI(4,5)P_2_ (167). In photoreceptor cells, PI(4,5)P_2_ regulates the biogenesis of light-sensing organelles and plays an important role in the delivery of rhodopsin-containing membranes to the ROS (33).

One of the receptor tyrosine kinases, epidermal growth factor receptor, has a cluster of basic residues in the juxtamembrane domain. PI(4,5)P_2_ directly binds to this region and has been shown to activate EGFR (168). These basic residues are also present in other growth factor receptors, such as IGF-1R, IR, FGFR1, PDGFR, VEGFR1, EPHB2, TRKA, and TRKB receptor (168). However, the activation of these receptors by PI(4,5)P_2_ has not been studied.

## Phosphatidylinositol-3-phosphate 5-kinase or pikfyve

In humans, FYVE finger-containing phosphoinositide kinase is encoded by the *PIKFYVE* gene (169). PIKfyve phosphorylates PI(3)P to PI(3,5)P_2_ and PI to PI(5)P (170, 171, 172). The FYVE finger domain of PIKfyve tethers to membrane PI(3)P and is essential for membrane localization of PIKfyve to the cytosolic leaflet of endosomes (173). This binding is needed to phosphorylate PI to PI(5)P and PI(3)P to PI(3,5)P_2_ (170). Dysregulated enzyme activity of PIKfyve results in enlarged lysosomes due to defective synthesis of PI(3,5)P_2_, which results in defective lysosome fission events; thus, PIKfyve navigates all aspects of the vesicular and endocytic pathways (172, 174). Mutations in one of the alleles of PIKfyve are linked to Francois-Neetens corneal fleck dystrophy (175). The ablation of both alleles in the mouse is lethal (176). Changes in PIKfyve have been shown to inhibit insulin-mediated glucose uptake (177). Mice with selective gene ablation of PIKfyve in skeletal muscle show prediabetic symptoms, which include insulin resistance, hyperinsulinemia, glucose intolerance, and increased adiposity (178). In one study, inhibition of PIKfyve resulted in the prevention of myocardial apoptosis and cardiac hypertrophy mediated through the activation of sirtuin-3 (*SIRT3*), a major mitochondria NAD^+^-dependent deacetylase in obese mice (179). Because photoreceptor cells are highly metabolic (180, 181), the PIKfyve enzyme might regulate several important functions. However, there are no available studies of this enzyme in the retina.

It was previously reported that Vps34-generated PI(3)P regulates the canonical autophagy (182). However, Vps34-independent noncanonical autophagy has been observed in sensory neurons from the PI(3)P-generated Vps34 enzyme, T-lymphocytes, and glucose-starved cells incubated with PI3K inhibitor, Wortmannin, which inhibits the Vps34 enzyme (183). PI(5)P-dependent noncanonical autophagy has been reported in cells depleted of PI(3)P (170). PIKfyve plays an important role in generating PI(5)P upon binding to PI(3)P through its FYVE domain (170). This PI(5)P can be converted to PI(4,5)P_2_ through the action of the type II phosphatidylinositol 5-phosphate 4-kinase (PIP4K) enzyme, which was previously shown to express in rod photoreceptor cells (163). The generated PI(4,5)P_2_ can perform various functions, including serving as a substrate for class I PI3K for the generation of PI(3,4,5)P_3_ (10, 80, 145). In cone photoreceptor cells, the deletion of class I PI3K that makes PI(3,4,5)P_3_ resulted in age-related cone degeneration (79, 143).

## Neuroprotective roles of PI3K in the retina

Several growth factors, such as PDGF, brain-derived neurotrophic factor, insulin, IGF-1 and -2, basic fibroblast growth factor, erythropoietin (EPO), and ciliary neurotrophic factor (CNTF), promote retinal cell survival through PI3K/Akt activation. Insulin and IGF-1 have been shown to promote photoreceptor survival (106, 184), whereas PDGF, CNTF, and EPO promote the survival of ganglion cells and RPE (185). basic fibroblast growth factor-mediated activation of PI3K has been observed in Müller cells (186). PI3K exerts its neuroprotective effect through its downstream effector, Akt (66, 106, 112). IR activity is important for PI3K/Akt activation (66, 106). For the signal to be maintained for a longer period, the protein tyrosine phosphatase, PTP1B, must be inactivated. It has been shown that activated Akt phosphorylates PTP1B on serine 50 (187), which inhibits the activity of PTP1B to facilitate a positive feedback mechanism for IR/PI3K/Akt signaling. In diabetic retinopathy, PI3K activity is downregulated (188) due to increased PTP1B activity (107). Furthermore, decreased activation of mTOR and p760S6K, and increased activation of GSK3β, the downstream effector of PI3K, are downregulated in diabetic retinopathy (189).

PI3K activation through the IR inhibits caspase-mediated cell death of retinal neurons in culture (190). 17β-Estradiol protects stressed retina through PI3K activation (191, 192). The deletion of the phosphatase and tensin homolog (PTEN) results in an increased level of PI(3,4,5)P_3_, which protects ganglion cells (193). Activation of PI3K through PDGF stimulation protects retinal pericytes under diabetic conditions (194). Activation of PI3K/Akt protects the RPE from oxidative stress (195, 196, 197). brain-derived neurotrophic factor-induced PI3K activation protects axotomized retinal ganglion cells (198). The neuroprotective function of CNTF is mediated through the PI3K pathway in vitro and in vivo (199). The PI3K/Akt pathway activated through cytokine EPO promotes adult CNS neuron regeneration (200). The PI3K pathway is necessary for nerve regrowth in the goldfish retina (201). PI3K also plays an important role in retinal development and survival of differentiated neurons in vivo (202). In addition, PI3K regulates the circadian output in the retina (203).

Constitutive activation of Akt has been observed in cone photoreceptor cells (204). In rods, Akt activation is transient under physiological light (66), hyperosmotic stress (205), oxidative damage (206), or light stress (112). In cone photoreceptor cells, PI metabolism is Ca^2+^-dependent and essential for glutamate release and synaptic ribbon formation (207). In dark-adapted cones, a rise in intracellular Ca^2+^ increases PI metabolism at the synaptic terminals compared with the ROS (207). These changes in cones result in the release of glutamate from the synapse, which halts PI metabolism in the adjacent horizontal cells. Under light-adapted conditions, increased PI metabolism has been observed in outer segments that support phototransduction (207). Decreased intracellular Ca^2+^ results in decreased PI metabolism in the synapse, which blocks the release of glutamate that releases the block on PI metabolism in the adjacent horizontal cells (207).

Inositol polyphosphates also play an important role in synaptic ribbon disassembly (207). Previous studies showed that the activation of PI metabolism and photoreceptor synapses under dark-adapted conditions is in parallel with the disappearance of synaptic ribbons (207). Further, inositol polyphosphates (IP_3_) have been shown to influence synaptic ribbons in cone photoreceptors but not in rods, suggesting that the cones have an active PI metabolism in cone synapses (207). Consistent with these studies, cones lacking class I PI3K exhibit disorganization of synaptic ribbons (207). In the retina/photoreceptors, a cross-talk exists between IR/PI3K and phototransduction. The PI3K pathway is activated through photobleaching of rhodopsin but is not dependent on G protein transducin activation (52). These observations suggest that other G proteins might be involved in the activation of the PI3K pathway. Such a cross-talk between tyrosine kinases and GPCR signaling has been observed outside of the retina. Mitogen-activated protein kinases, extracellular-regulated kinases, stress-activated protein kinase (208, 209), and nonreceptor protein tyrosine kinases (210) are the best examples of this cross-talk.

## Phosphoinositide phosphatases

To date, over 35 mammalian phosphoinositide phosphatases that downregulate many phosphoinositide signals have been identified (211). A retina proteome shows the presence of phosphoinositide phosphatases that are encoded by Mtmr2 and Synj1, Inpp1, Inpp4a, Inpp4b, and Inpp5e genes (162). Synaptojanins regulate synaptic function (212, 213, 214), which includes endocytosis and vesicle uncoating, and profound cone defects have been observed in zebrafish when this phosphatase is lacking (215, 216, 217). In cone photoreceptor cells, the phosphoinositide phosphatase, synaptojanin 1 (SynJ1), has been shown to regulate autophagy and endosomal trafficking, and mutations in zebrafish cone photoreceptors show abnormal accumulation of late endosomes and autophagosomes (215, 217, 218, 219). Synaptojanin 1 has also been shown to regulate cognition and plays an important role in Down syndrome (220).

Defects in oculocerebrorenal syndrome of Lowe (OCRL) phosphatase are associated with glaucoma, a second leading cause of blindness worldwide (221, 222). Mutations in inositol polyphosphate 5-phosphatase (INPP5)E are associated with Joubert syndrome and Bardet-Bedi syndrome (223, 224, 225, 226), which result in retinal degeneration. Both PI(4,5)P_2_ and PI(3,4,5)P_3_ serve as substrates for INPP5E and the levels of PI(4,5)P_2_ are suggested to be important for the regulation of cilium (14). The 3′phosphatase PTEN regulates retinal neurogenesis and Notch signaling (227). Further inactivation of PTEN in the RPE causes retinal degeneration, highlighting the importance of this phosphatase in RPE functions (228). In an animal model of retinitis pigmentosa, the deletion of PTEN in cone photoreceptors resulted in cone photoreceptor survival (229). The retinal amacrine cell number is shown to be regulated by PTEN through the modulation of Erk, TGFβ, and serine-threonine kinase Akt (230). Retinal interneuron morphogenesis and synaptic layer formation are shown to be regulated by PTEN (231). Interestingly, the deletion of PTEN together with the retinoblastoma gene resulted in penetrant bilateral retinoblastomas (232). PI3K activation triggered by insulin or other growth factors phosphorylates PI(4,5)P_2_ to PI(3,4,5)P_3_, which can be rapidly dephosphorylated by PTEN to generate PI(4,5)P_2_ or by INPP5s to generate PI(3,4)P_2_. INPP5 also hydrolyzes PI(4,5)P_2_ to PI(4)P (211).

In mammalian cells, ten 5-phosphatases have been identified. These 5-phosphatases regulate several aspects of cellular function, including synaptic vesicle recycling, cell proliferation, embryonic development, control of hematopoietic development, and insulin-mediated signaling (233). The 5-phosphatases, OCRL and INPP5E, are mutated in Lowe and Joubert syndrome, respectively (234). Src homology 2 domain-containing inositol polyphosphate 5-phosphatase (SHIP)2 and skeletal muscle- and kidney-enriched inositol phosphatase (SKIP) are negative regulators of insulin signaling and glucose metabolism (235, 236). Insulin resistance is associated with polymorphisms in SHIP2 (237). INPP4A and INPP4B, the inositol 4-phosphatases, dephosphorylate PI(3,4)P_2_ to PI(3)P, and these phosphatases have been shown to control neuroexcitatory cell death (238, 239). The Sac phosphatases dephosphorylate PI(3)P, PI(4)P, PI(5)P, and PI(3,5)P_2_ to form PI (211). Degenerative neuropathy has been reported with loss of one of the Sac phosphatase genes, FIG4 gene (240). Like phosphoinositide kinases, mutations or changes in the expression of phosphatases cause many human diseases (211).

## The role of phosphoinositides in the invertebrate retina

Although this review focuses on phosphoinositide metabolism in the vertebrate retina, it is important to note that PI metabolism is also important in the invertebrate retina. Visual excitation in *Limulus* (horseshoe crab) photoreceptors have been shown to be mediated by *myo*-inositol polyphosphate (241). In *Drosophila* photoreceptor cells, light stimulated the hydrolysis of PI(4,5)P_2_ by PLC into DAG and IP_3_ (242, 243, 244). IP_3_ facilitates the opening of two calcium-sensitive channels, TRP and TRP-like (218), which results in plasma membrane depolarization (245). In the vertebrate retina, cGMP modulates the channel but not the IP_3_ (26). In squid photoreceptor membranes, PI-PLC is activated through metarhodopsin-activated G protein, Gq (246). In invertebrates, retinal degenerations result from a mutation in various enzymes of the PI cycle (243, 244, 245). In *Drosophila*, phosphatidylinositol transfer protein (PITP) is essential for photoreceptor cell survival and recovery of light stimulation (250). Further, rhodopsin inactivation is mediated by the visual arrestin, Arr2. Its translocation from the rhabdomeric cell body to the microvilli is governed by myosin III-driven PI(3,4,5)P_3_-dependent movement (251, 252, 253, 254). *Drosophila* Arr2 has a PH domain that binds to PI(3,4,5)P_3_ (251), but mammalian visual arrestins do not. The light-induced trafficking of Arr2 to the microvilli, the termination of phototransduction, and recovery are impaired in *Drosophila* retinas lacking phosphoinositide biosynthesis and trafficking genes *cds* and *rdgB* or overexpression of 3′phosphoinositide phosphatase PTEN, which dephosphorylates PI(3,4,5)P_3_ to PI(4,5)P_2_ (255). In *Drosophila*, light-activated TRP and TRP-like channels regulate the PI signaling cascade (256). In *Drosophila* photoreceptor membranes, PI(4)P synthesis is catalyzed by PI4KIIIα (257). Further, PIP5K is needed for the generation of PI(4,5)P_2_ for GPCR signaling in *Drosophila* (258).

## Roles of phosphosphoinositide lipids in the retina

Alterations in the metabolic flow of events in the retina can lead to retinal degeneration (see RETNET; https://sph.uth.edu/retnet/, May 2020). It has been reported that blue light-excited all-*trans*-retinal and 11-cis-retinal irrevocably disrupt plasma membrane-bound PI(4,5)P_2_ and alter its function in nonvisual cells (259). This altered PI(4,5)P_2_ increases cytosolic calcium and causes a change in cell morphology, leading to cell death (259). These studies suggest photoexcited chromophore-prompted PI(4,5)P_2_ alteration, which leads to oxidative damage to the plasma membrane (259). This altered PI(4,5)P_2_ function is independent of nonvisual GPCR activation (259). mTOR1 activation is mediated by lysosomal positing, while amino acids promote the recruitment of FYVE and coiled-coil domain autophagy adaptor 1 (FYCO1), a PI(3)P binding protein, to the lysosomes and facilitate the interaction between FYCO1 lysosomes and the ER that encompasses protrudin, a PI(3)P effector protein (260). These studies suggest that the Vps34-generated PI(3)P regulates mTORC1 activation through lysosomal positioning (260). In addition, the cause of congenital cataracts is due to mutations in the FYCO1 gene (261). In the retina, type 1 metabotropic glutamate receptors are coupled to the hydrolysis of polyphosphoinositide (262). Class I PI3K in the retina has a dual function in regulating cell survival and cell trafficking (263). A mathematical model of the PI signaling pathway has been constructed, and the authors argue that this model reflects the study state of the PI pathway (264). In the zebrafish retina, PITPβ is needed for the integrity of cone cell outer segments (265). PITP protein transfers the PI and PC across membranes (266). Further, mutations in the PYK2-binding domain of phosphatidylinositol transfer membrane-associated protein (PIPNM3) caused autosomal dominant cone dystrophy (CORD5) in two Swedish families (267). The cone CNG (31) and olfactory (32) channels are known to be inhibited by the PI3K-generated PI(3,4,5)P_3_. There are no available studies on the role of PI(3,4,5)P_3_ in rod CNG channels. Further, PLC and PI(4,5)P_2_ levels in phototransduction are regulated by ceramide kinase (268). The components of PI signaling in photoreceptor outer segments were identified in the mid-1990s (25). PIPs and proteins that are involved in phosphorylation of these lipids have been observed in the axoneme of the bovine ROSs (269). In ROSs, transducin βγ-subunits have been shown to regulate the metabolism of PI(4,5)P_2_ (270). Alterations in retinal structure and PI degradation by light damage and chronic lithium treatment have been reported (271).

The PI synthesis in photoreceptor cell inner segments is enhanced by light and cytidine (20, 272). In rat retina, the PI synthesis and phosphorylation are stimulated by light (41). The PI turnover is also enhanced in light (21). The lipid PI(4,5)P_2_ has been shown to regulate blood vessel stability; this lipid could be a viable therapeutic target for angiogenesis therapies in diseases such as AMD, cancer, and proliferative diabetic retinopathy (273). RPE-mediated photoreceptor phagocytosis is regulated by phosphoinositide lipid signaling (274). In cone photoreceptor cells, CNGA3-associated mutation potentiates the PI sensitivity of cone-CNG channels by disrupting the interaction between subunits (275). In photosensitive chicken retinal ganglion cells, light has been shown to stimulate the PI cycle (276).

## Phosphoinositides in retinal cilia

PI(4)P and PI(4,5)P_2_ are important for the regulation of primary cilia, especially in assembly and disassembly (277, 278, 279, 280, 281). Phosphoinositide signaling has been coupled with proper protein trafficking and Hedgehog signaling in the primary cilia (280, 282). The 5′PI phosphatase, INPP5E, is paired with proper ciliogenesis (280, 282).

INPP5E dephosphorylates PI(3,4,5)P_3_ to PI(4,5)P_2_ (282). This inositol phosphatase is important in ciliary development in zebrafish (223). Mutations in INPP5E result in Joubert syndrome, a rare disorder characterized by deformation of the midbrain, retinitis pigmentosa, renal cysts, and polydactyly (283). INPP5E has been shown to regulate the phosphoinositide-dependent cilia transition zone function. IPP5E mutant embryos have dysfunctional Hedgehog signaling (284). In the retina, ciliopathy protein RPGR interacts with phosphodiesterase 6δ and regulates the ciliary localization of INPP5E (285).

In addition to INPP5E, OCRL is also important for the regulation of cilia (277, 278, 279, 280, 281, 286). Bardet-Biedl syndrome is caused by the mutations in BBSome, and this protein complex is involved in ciliary trafficking and binds to phosphoinositides (287, 288). In vitro, the PH domains of BBS5 bind to PI(3)P (287). Similar specificities in PIP binding have been observed with BBS1, -4, -5, -8, -9, and -18. A recent review of phosphoinositides mentioned the caveat that commercial PIP strips have local surface densities of PIPs, which are significantly different under physiological conditions (14). Further studies are warranted to establish that these proteins indeed bind to PIPs in vivo.

## The signaling role of phosphoinositides in a cellular process

The phosphorylated phosphoinositides are formed in response to growth factor stimulation or GPCR activation or are activated by other cellular cues that attract proteins from the intracellular side of the cells through protein-lipid interactions. The proteins that interact with PIPs are called phospholipid-binding or phosphoinositide binding proteins. It is essential to establish the specific lipid involved in given cellular processes. It is also important to identify the effector protein that is regulated by the PIP signal that governs the cellular functions.

Investigators have identified several phosphoinositide-binding proteins that relay the PIP signal. Generally, two groups of interacting sites exist for the stereoselective association of PIP with effector proteins (247). The first group comprises a 10–20 co-linear sequence enriched with basic and hydrophobic residues that mediate interaction with PIP (247). The second group has proteins consisting of around 120 amino acids that share homology with a well-established tertiary structure of the PH domain (247). These PH domains are extensively characterized and are well-known PIP binding modules. The PH domain sequence was first observed in the platelet PKC substrate pleckstrin (248), which contains a collinear sequence of 120 amino acids and is present in more than 100 proteins.

PI(3)P binds to FYVE and PX domain-containing proteins (247). PI(4,5)P_2_, PI(3.4)P_2_, and PI(3,4,5)P_3_ bind to proteins containing PH domains (249, 289, 290, 291). PI(5)P binds to proteins containing the plant homeodomain domain (292). Several studies showed that phosphoinositides were detected qualitatively or quantitatively using the PH, PX, and FYVE domains (291, 293). The FYVE domains of EEA1 and Hrs have been used to detect PI(3)P (173, 291, 294). The PH domains of FAPP1, OSBP, and OSH2 and the P4M domain of SidM/GOLPH3 have been used to detect PI(4)P (291). The plant homeodomain domain of ING2 (292), Tam1 DH-PHc domain (295), and the VHS domain of TOM1 have been used to detect PI(5)P (296). The PROPPIN domain of Atg18P and the WD40 domain of raptor have been used to detect PI(3,5)P_2_ (297). The PH domains of TAPP1, p47phox, and PKB/Akt have been used to detect PI(3,4)P_2_ (291). The PH domain of PLCδ1 and the PX domain of tubby have been used to detect PI(4,5)P_2_ (291). The PH domains of PKB/Akt, GRP1, Btk, and ARNO have been used to detect PI(3,4,5)P_3_ (249, 291). Some of these domains may bind to more than one PIP lipid (291).

**Fig. 5:**
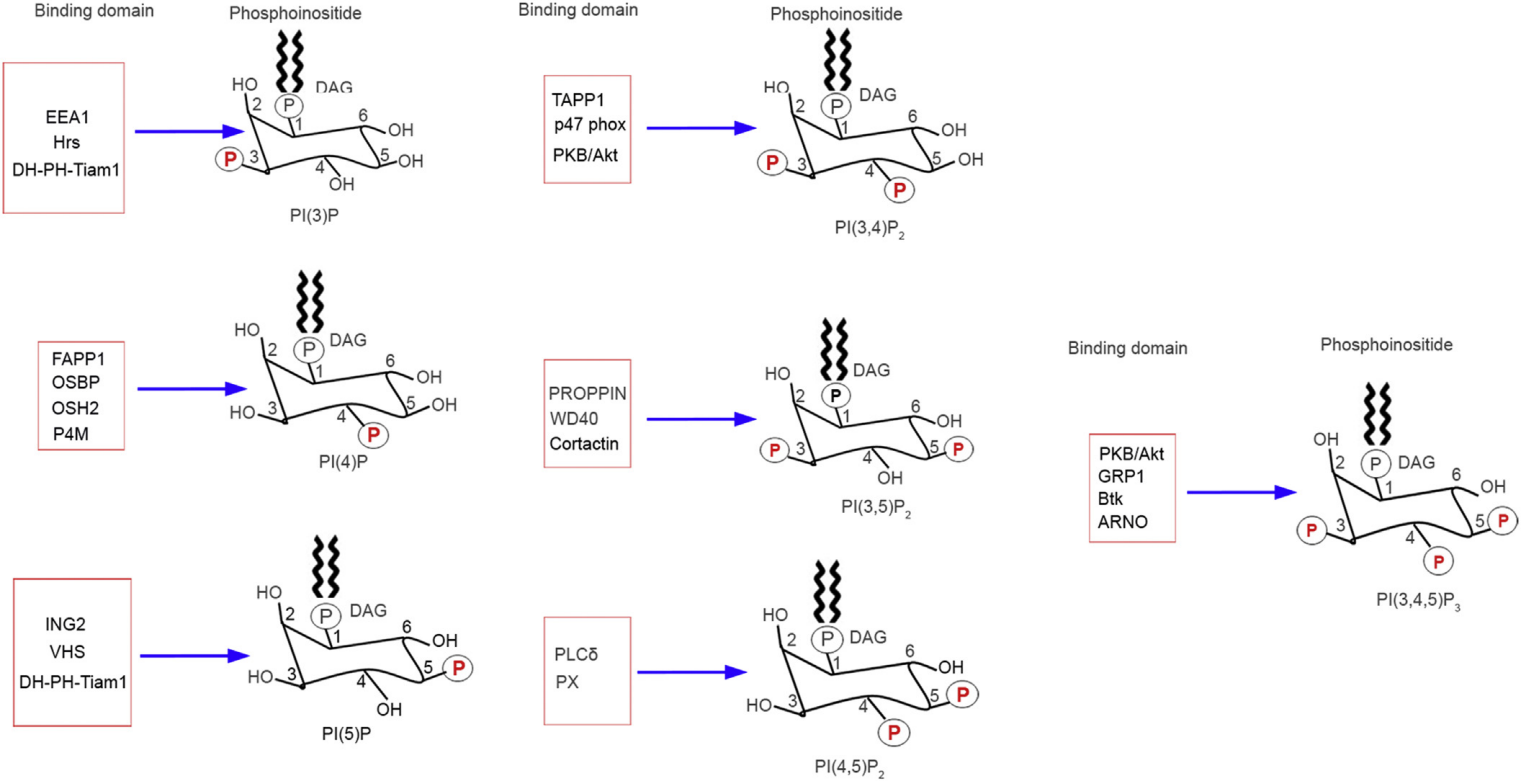
Commonly used phosphoinositide-binding protein probes (domains) to determine the levels and localization of specific PIPs in vitro and in vivo.

Cortactin has been shown to bind to PI(3,5)P_2_ (298). It does not contain canonical PIP binding motifs, such as PH or FYVE, but contains a stretch of basic lysine residues that may bind to PIP. This region is located within the N-terminal fourth repeat domain of cortactin (298). One study showed that the lactadherin C2 domain binds to PS (299); this domain could be used as a probe to determine the levels of PS in cells/tissues. PH domains do not always bind to PIPs, but sometimes act as platforms for protein-protein interaction domains (300, 301). Recently, the PIP binding domain has been used to quantitate the levels of PI3K-generated PIs in rod photoreceptor cells (53). A list of phospholipid-binding proteins that bind to specific PIPs commonly used as probes to study protein-lipid interaction is presented in Fig. 5.

## Methods to detect cellular levels of phosphoinositides

Mass spectrometry is commonly used to detect PIPs. The PIPs can be identified through the *m/z* values of their collision-induced fragments. This approach provides a “fingerprint” for each PIP (3). By including a standard such as lysophosphatidic acid, the specific PIP can be identified and quantitated (3). Mass spectrometric methods are very sensitive and overcome the need for radioactive labeling.

Effector phosphoinositide binding proteins bind to specific PIPs. The proteins fused to reporters have been used as valuable tools to analyze specific PIPs in live or fixed cells and tissues (3, 53). Fluorescently tagged phosphoinositide-binding protein (either GFP or DsRed) or antibodies against fusion proteins have been used as probes for the immunoelectron or fluorescent microscopic detection of specific localization of PIPs (302). In rod photoreceptor cells, RPE cells, zebrafish, and transgenic *Xenopus* retina, PIP probes have been successfully employed to determine the localization of specific PIPs (53, 66, 100, 217, 218).

The fastest way of testing the potential phosphoinositide binding proteins is through protein-lipid overlay assays using purified phosphoinositide-binding proteins in vitro (290, 293, 299). Because the PIPs are localized to membranes, sometimes they may not interact with a specific phosphoinositide-binding protein in vitro. In those cases, liposomes containing specific PIPs can be used to mimic cellular membranes (3). Incubation of these liposomes with phosphoinositide-binding protein and the associated phosphoinositide-binding proteins with liposomes can be recovered by centrifugation of heavy multilamellar liposomes or affinity isolation of small unilamellar vesicles, followed by immunoblotting or radioactive counting (3). Liposome-based assays are better in terms of measuring differential affinities of phosphoinositide-binding proteins to PIP than are protein-lipid overlay assays (3). These liposomes can be placed on the surface of the sensor chip for surface plasmon resonance spectroscopy (3). Association and dissociation constants can be calculated when the phosphoinositide-binding proteins are passed through a flow cell and bind to the immobilized PIP, which causes a change in surface refractory index (3).

Identification and measurement of phosphoinositide-metabolizing enzyme activity is another way of measuring PIPs in a given tissue or cell. These assays can be performed using radioactive ATP followed by TLC, after which the incorporated radioactivity in the PIPs can be counted (52, 104, 303). Nonradioactive enzyme assays are also available to determine the phosphoinositide-metabolizing enzymes (304). Many assays have been developed for the measurement of PI kinases and PI phosphatases in cell extracts or with purified proteins. These extracts or purified proteins can be incubated with radiolabeled PIPs, and their phosphorylated or dephosphorylated PIPs can be monitored using TLC or HPLC (305). Researchers have also developed novel mass assays to quantify phosphoinositides from cells and tissues (306, 307, 308).

Competitive ELISAs have been developed to determine the PI kinase assays (3). Several ELISAs have been developed to quantify the levels of specific PIPs using phosphoinositide-binding proteins (249, 309). The phosphoinositide-binding proteins are coupled to enzymes, peroxidase-conjugated antibodies, or antibody epitopes for the detection and quantification of various PIPs in cells and tissues (53, 309, 310). In photoreceptor cells, PI3K-generated products have been shown using phosphoinositide-binding proteins coupled with rhodopsin 1D4 antibody epitope on ELISA (53, 311). Fluorescence resonance energy transfer is another technique to determine the binding between PIP and phosphoinositide-binding proteins (3).

There are many other high-resolution techniques coupled with molecular probes to visualize the location, organization, and dynamics of PIPs (312). These techniques include single-particle tracking, fluorescence recovery after photobleaching, and fluorescence correlation spectroscopy (313). The combination of lipid probes and available microscopic techniques is very powerful, but these approaches have several potential limitations and caveats (313). There are numerous commercially available PIP-specific antibodies. A number of investigators have used these antibodies to localize PIP in cells and tissues (54, 314, 315, 316). Also, transgenic frogs (66) and zebrafish (217) models have been used to study the generation and localization of PIPs in vivo.

## Conclusions and future directions

Numerous studies have explored the roles of various phosphoinositides, phosphoinositide kinases, and phosphoinositide phosphatases in the vertebrate retina. The minor lipids play an important role in various aspects of photoreceptor biochemistry and physiology. These lipids are involved in protein transport, ciliogenesis, cell survival, vesicular transport, autophagy, phagocytosis, and synaptic functions. Many researchers have demonstrated that dysfunctional PI metabolism causes retinal degenerative diseases. In other cell types, various PIPs are generated through the activation receptors. However, in the retina, light stimulates the activation of phosphoinositide-metabolizing enzymes. The mechanism of these enzyme activations is currently under investigation. Future studies should be aimed at the function and mechanism of activation of phosphoinositide-metabolizing enzymes/phosphatases and PIP function and regulation. The phosphoinositide metabolism in the cone synapse is different from that of rod synapses. Identification of phosphoinositide kinases and phosphoinositide phosphatases and identification and localization of specific PIPs in various cell types of the retina in health and disease would advance the field.

## Conflict of interest

The author declares that he has no conflicts of interest with the contents of this article.
